# N-glycans of the microalga *Chlorella vulgaris* are of the oligomannosidic type but highly methylated

**DOI:** 10.1038/s41598-018-36884-1

**Published:** 2019-01-23

**Authors:** Réka Mócsai, Rudolf Figl, Clemens Troschl, Richard Strasser, Elisabeth Svehla, Markus Windwarder, Andreas Thader, Friedrich Altmann

**Affiliations:** 1 Department of Chemistry, University of Natural Resources and Life Sciences, Vienna; Muthgasse 18, 1190 Vienna, Austria; 2 Department of Agrobiotechnology Tulln, University of Natural Resources and Life Sciences, Vienna; Konrad-Lorenz-Straße 20, 3430 Tulln an der Donau, Austria; 3 Department of Applied Genetics and Cell Biology, University of Natural Resources and Life Sciences, Vienna; Muthgasse 18, 1190 Vienna, Austria; 4 Present Address: Fresenius Medical Care Adsorber Tec GmbH, Magnesitstraße 9, 3500 Krems, Austria; 5 Present Address: Shire, Process Development & Technical Services, Benatzkygasse 2-6, Vienna, Austria; 6 Present Address: IST Austria, Am Campus 1, 3400 Klosterneuburg, Austria

## Abstract

Microalgae of the genus *Chlorella vulgaris* are candidates for the production of lipids for biofuel production. Besides that, *Chlorella vulgaris* is marketed as protein and vitamin rich food additive. Its potential as a novel expression system for recombinant proteins inspired us to study its asparagine-linked oligosaccharides (N-glycans) by mass spectrometry, chromatography and gas chromatography. Oligomannosidic N-glycans with up to nine mannoses were the structures found in culture collection strains as well as several commercial products. These glycans co-eluted with plant N-glycans in the highly shape selective porous graphitic carbon chromatography. Thus, *Chlorella vulgaris* generates oligomannosidic N-glycans of the structural type known from land plants and animals. In fact, Man5 (Man_5_GlcNAc_2_) served as substrate for GlcNAc-transferase I and a trace of an endogenous structure with terminal GlcNAc was seen. The unusual more linear Man5 structure recently found on glycoproteins of *Chlamydomonas reinhardtii* occurred - if at all - in traces only. Notably, a majority of the oligomannosidic glycans was multiply *O*-methylated with 3-*O*-methyl and 3,6-di-*O*-methyl mannoses at the non-reducing termini. This modification has so far been neither found on plant nor vertebrate N-glycans. It’s possible immunogenicity raises concerns as to the use of *C*. *vulgaris* for production of pharmaceutical glycoproteins.

## Introduction

Chlorella is a well-known member of the taxonomically enormously diverse group of microalgae. It enjoys considerable attention as a production system for various lipids, either as biofuel source^1–5^ or as food and feed additives such as carotenoids or astaxanthin^6,7^. *C*. *vulgaris* and “*C*. *pyrenoidosa*” - a still applied but unfortunately outdated classification^8,9^ - are offered as dietary supplements with diverse assertions of health benefits^10,11^. Chlorella species are also being studied as production platforms of recombinant proteins^12–14^. This warrants interest in the potential of Chlorella and other microalgae used for this purpose to conduct post-translational modifications, in particular protein glycosylation.

High-mannose N-glycans have been found in various microalgae such as the diatom *Phaeodactylum tricornutum*^15^ but also unusual structures such as 6-*O*-methyl mannose on the red alga *Porphyridium* – a taxonomic group rather unrelated to green plants - have been discovered^16^. Euglena – sometimes referred to as microalga – but actually a member of a separate phylum (or group) to which *i*.*a*. the trypanosomes belong – were found to contain oligomannosidic structures partially modified with aminoethylphosphonate moieties^17^. The green alga *Chlamydomonas reinhardtii* was reported to produce glycoproteins with mammalian-type N-glycans even containing sialic acids^18^. Later, *C*. *reinhardtii* was shown to contain oligomannose glycans – or better low-mannose glycans due to their limited size of hardly more than 5 mannoses - but also glycans with up to two xylose residues and with 6-*O*-methyl mannose^19^. A glycopeptide based study on *Botryococcus brauni* – a green algae belonging to the class of *Trebouxiophyceae* just as Chlorella - discovered N-glycopeptides with up to three GlcNAc residues indicating action of GlcNAc-transferase I (GnTI)^20^. Furthermore, methyl-hexose and pentose were found by CID-MS/MS of glycopeptides. This small number of papers on glycoprotein structures of microalgae (including just a few green algae) can be rated as a sign of ignorance given their ecological significance and their growing role as biofactories. In particular, *C*. *reinhardtii* and *Chlorella* species are regarded as promising production hosts for proteins and glycoproteins^14,21^ and the diatom microalgae *Phaeodactylum tricornutum* has recently been demonstrated to produce a fully functional anti-hepatitis antibody with high-mannose glycans^22^. However, in the green alga *C*. *reinhardtii* the Man_5_GlcNAc_2_ N-glycan assumed to represent a substrate for recombinant GnTI turned out as having an unusual, more linear structure inaccessible for GnTI^23^.

In this work we investigated the N-glycosylation of *Chlorella vulgaris* strains from culture collections as well as of commercial products. MALDI-TOF MS, chromatography on graphitic carbon and amide silica, gas chromatography of constituent sugars and action of GlcNAc-transferase I (GnTI) were applied to characterize the N-glycans of *C*. *vulgaris*.

## Results

### MALDI-TOF MS profiles of *Chlorella vulgaris* N-glycans

**Figure 1:**
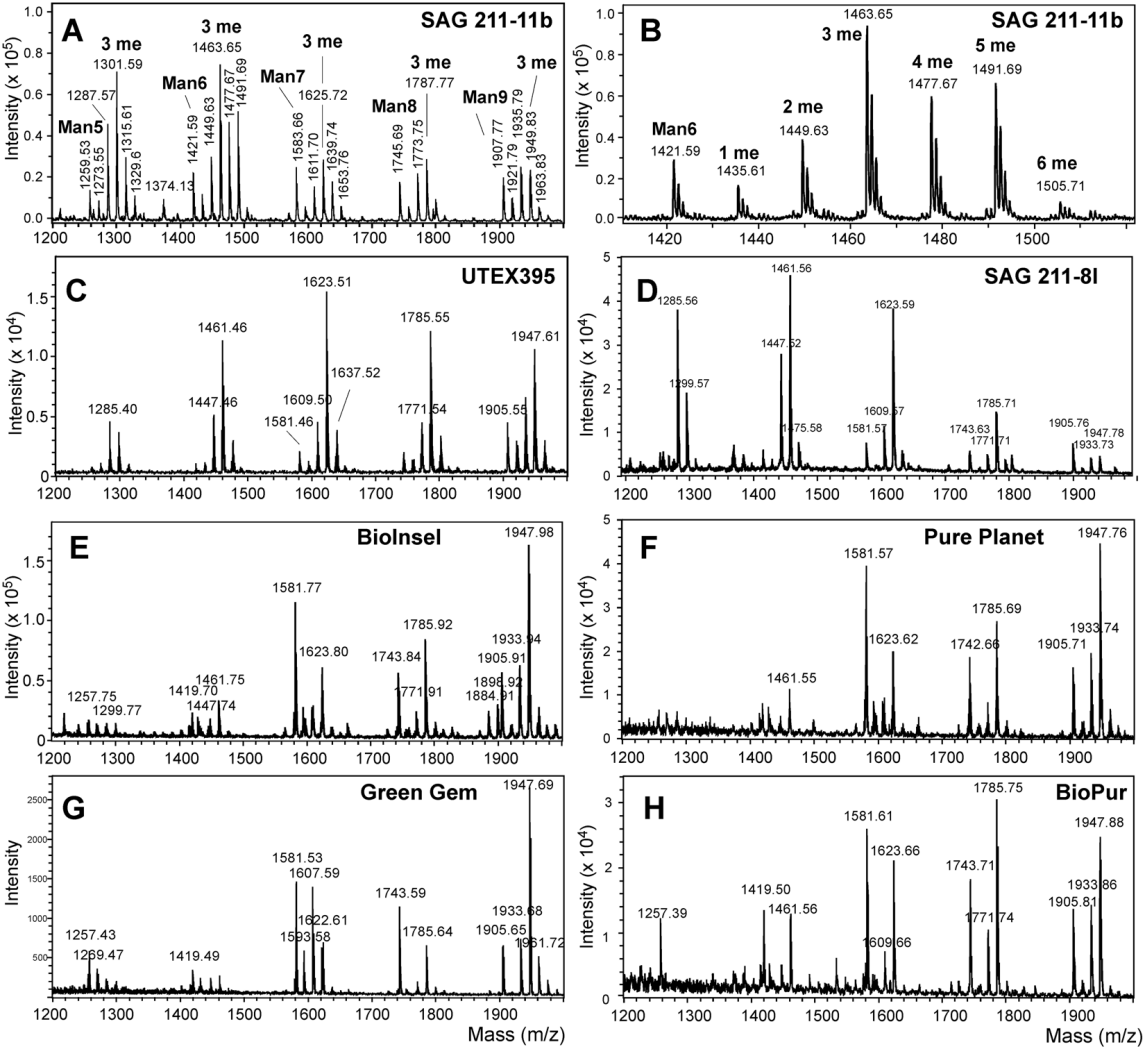
N-glycan profiles of *Chlorella vulgaris* culture collection strains and several commercial Chlorella products. MALDI-TOF MS patterns of reduced (panel A and B) and native N-glycans (all other samples) are shown for the culture collection strains *Chlorella vulgaris* SAG 211-11b and 211-8 l (**A**–**C**), and UTEX 395 (**D**) and for commercial products (panels E to H).

N-glycans from the live strain SAG 211-11b were isolated from the complete bulk material by a succession of pepsin digestion, cation exchange, PNGase A digestion and another cation exchange step. MALDI-TOF MS of the resulting oligosaccharides revealed a rather complex pattern with five prominent groups of peaks. The smallest masses within each group had compositions from Man_5_GlcNA_2_ to Man_9_GlcNAc_2_ (Man5 to Man9) (Fig. 1A). These well-known compounds were followed by peaks spaced by 14.018 Da indicating series of methyl groups (Fig. 1B). Essentially the same profiles were obtained when samples were repeatedly (>four times) analyzed as in the case of SAG 211-11b and GreenGem.

The same compounds albeit in different proportions were found in the *C*. *vulgaris* strains UTEX 395 (Fig. 1C) and SAG 211-8 l (Fig. 1D). Very similar MALDI-TOF MS patterns were found for several commercial *Chlorella* products (Fig. 1E–H). Remarkably, some of these strains were designated as *C*. *pyrenoidosa*, although this taxonomic name has been dismissed some time ago and the respective strains have been assigned as other *Chlorella* species and lines or even as other genera^8^. Many commercial products nevertheless bear this species name and it still occurs in the scientific literature. It shall not be concealed that other “*C*. *pyrenoidosa*” products exhibited different N-glycan profiles that apparently contained pentoses, *O*-methyl groups and possibly deoxyhexoses but these shall be subject of a future study.

### Location of methyl groups

**Figure 2:**
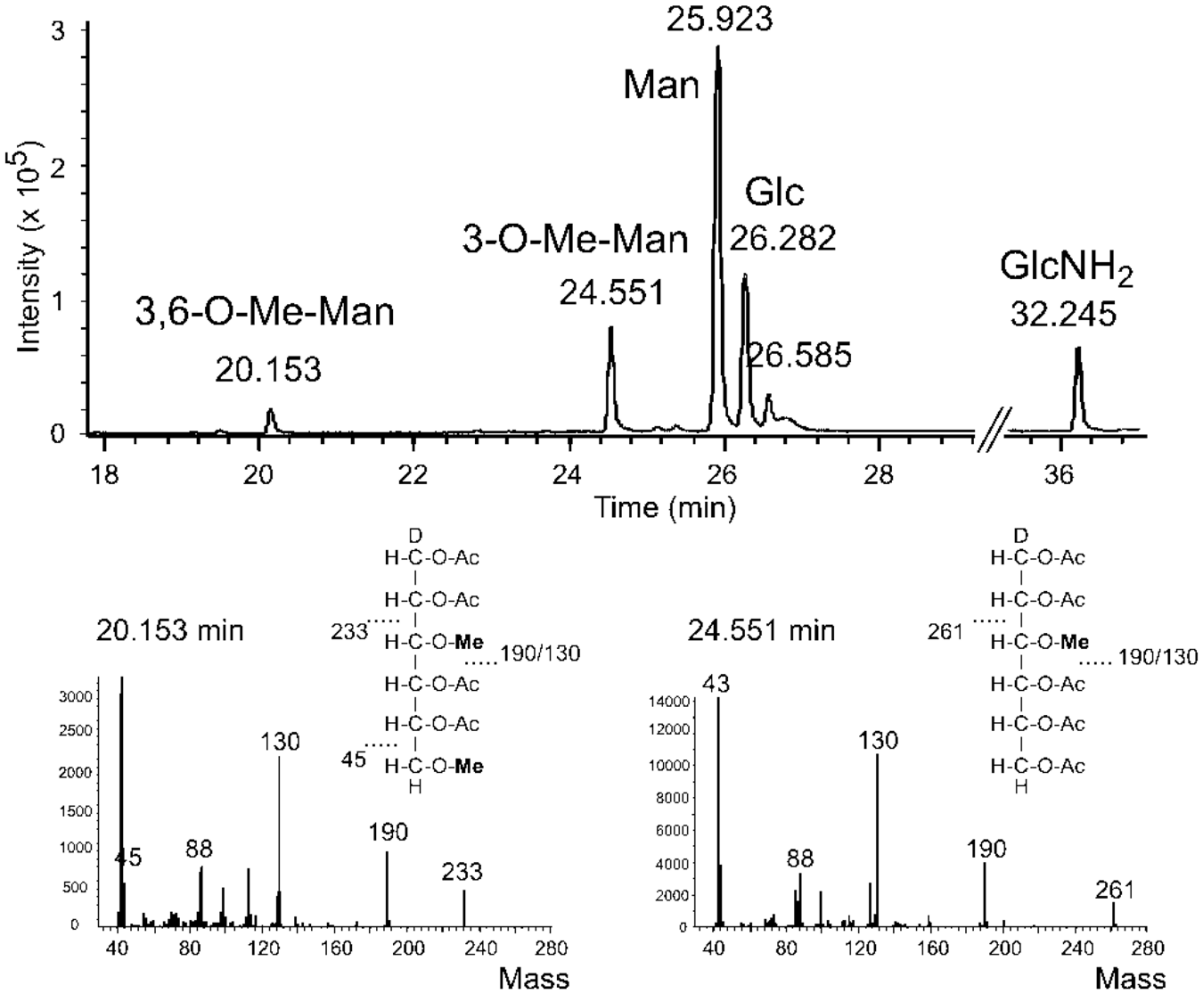
Constituent analysis of a *Chlorella vulgaris* 211-11b sample by GLC-MS. The bottom panels show the spectra for di- and mono-methylated hexose peaks, which were identified as 3,6-*O*-methyl mannose and 3-*O*-methyl mannose by their retention time.

To characterize the nature of glycan methylation, N-glycans from SAG211-11b and from GreenGem tablets were hydrolyzed. The monosaccharides were reduced, peracetylated and subjected to GC-MS together with suitable partially methylated standards^24^. This revealed the presence of 3-*O*-methyl mannose (24% compared to the mannose peak) and smaller amounts of 3,6-di-*O*-methyl mannose (4%) (Fig. 2) in *C*. *vulgaris* 211-11b. Similar values were found for the GreenGem sample, whereby the only semiquantitative nature of this figures shall be conceded as reference compounds for quantitative analysis of the methyl hexoses were not available.

**Figure 3:**
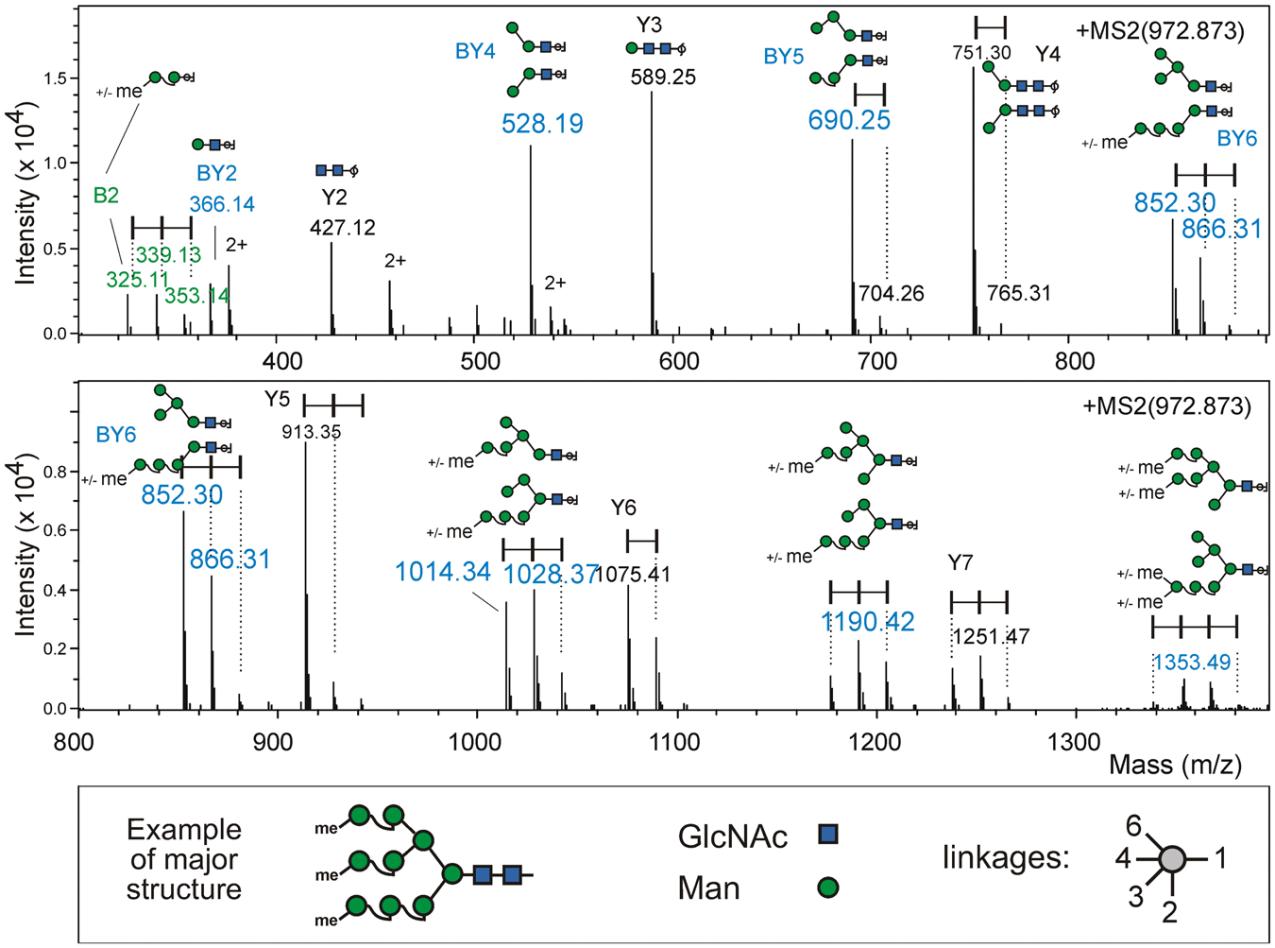
ESI-MS/MS spectrum of tri-*O*-methylated Man9. The spectrum is dominated by Y-ions and BY-ions lacking the reducing GlcNAc. Series of peaks spaced by 14.018 Da are bracketed. Cartoons show a selection of possible fragment structures.

**Figure 4:**
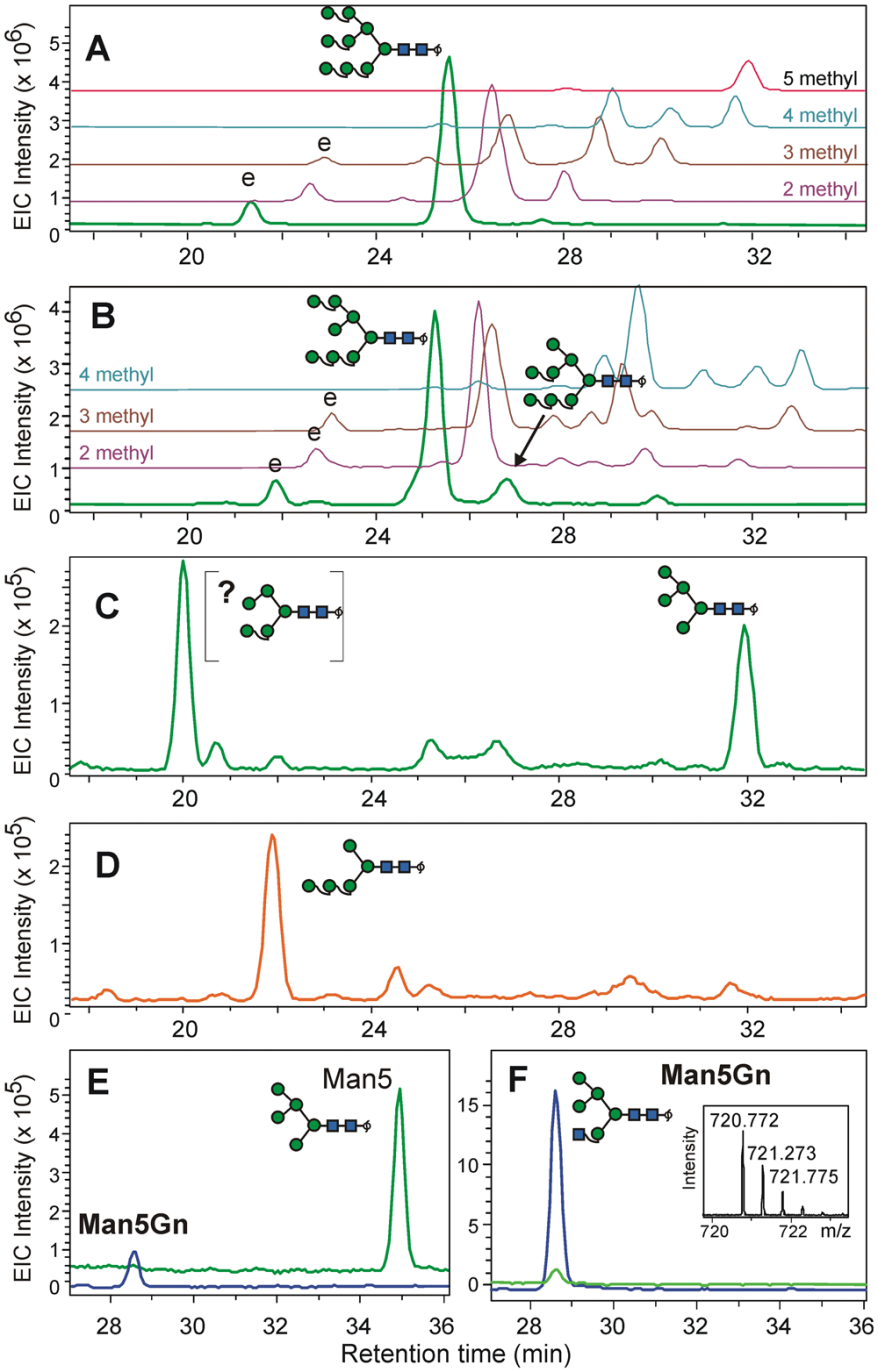
Analysis of *C*. *vulgaris* N-glycans by PGC-LC-ESI-MS. Panels (A,B) show the elution profiles of *Chlorella* glycans Man9 and Man8, respectively. Isomeric structures were deduced from coelution with bean N-glycans. The EIC traces of di- to penta-methylated glycans are shown in the background. Peaks labeled with “e” represent epimerization artefacts of the major peaks. Panel (**C**) is the EIC for *Chlorella* Man5 with an unusual early eluting peak in addition to the regular Man5 structure. Panel (D) gives the elution pattern of the Man5 isomer from an ALG3 deficient Arabidopsis line^27^. Panels (E,F) demonstrate the effect of GnTI on *Chlorella* Man5 in the absence (**E**) and presence (**F**) of UDP-GlcNAc.

MALDI-TOF LIFT MS/MS spectra of underivatized glycans showed little useful detail about the location of the methyl groups. In order to obtain ESI-MS/MS spectra without the risk of hybrid spectra with precursors of differing degree of methylation (7 Th mass difference at charge state 2), we attempted HILIC fractionation, which led to a preparation of suitably isolated trimethylated Man9. The ESI-MS/MS spectrum of Me_3_Man_9_GlcNAc_2_ was in perfect agreement with the assumption that all methyl groups were attached to terminal mannose residues (Fig. 3). Y-ions or GlcNAc-truncated y-ions showed +14 Da increments only at a size range that possibly contained terminal Man residues. The pattern is particularly consistent with the assumption of a major fraction with all terminal residues being mono-methylated and a minor part with a non-, mono- and dimethylated mannose each. In fact, the presence of at least three isomers of Me_3_Man_9_GlcNAc_2_ is indicated by PGC chromatography (Fig. 4A). As a detail out of many, the large dimethylated Man9 peak (Fig. 4A) generates B-ions for a mono-methyl-mannose m/z = 339.1), whereas the later eluting, smaller one that for a di-methyl-mannose m/z = 353.1).

Then again, the smaller glycans down to Man5 also were decorated with up to 4 methyl-groups. I.e. residues other than those modified in Man9 must have born the methyl groups. This insight has an interesting repercussion as to the biosynthesis of these glycans: The methyl groups are transferred to the mature N-glycans.

### Isomer structure of oligomannosidic N-glycans in *C*. *vulgaris* and detection of Man5Gn

The isomeric structures of oligomannosidic glycans of *C*. *vulgaris* SAG 211-11b by PGC-HPLC were studied by PGC-LC-ESI-MS. Comparison with the N-glycans of white kidney beans revealed co-elution of almost all non-methylated glycans as shown for Man8 and Man9 (Fig. 4). An EIC for the mass of Man5, however, gave two peaks, one at the position of the common (M^6^M^3^)M and another one eluting much earlier. At first, we verified the structure of the second peak by incubation of a HILIC enriched Man5 fraction with recombinant GnTI. The peak was converted to Man5Gn (Fig. 4B and Supplementary Fig. 1) and so elution time and transferase specificity argued for the classic (M^6^M^3^)M structure [for an explanation of nomenclature consult reference^25^]. Further support for this conclusion came from the accessilibity of the Man5Gn product to core α1,6-fucosyl transferase (Supplementary Fig. 2).

The earlier eluting peak Man5 that was not converted by GnTI may have the unusual structure with two α1,2-linked mannoses on the 3-arm but no branching mannoses on the 6-arm recently described as the only Man5 glycan in *Chlamydomonas reinhardtii*^23^. This MM^2^ isomer would elute rather early on PGC^25^. To verify its nature, we prepared glycans from an *alg*3 and also mannosidase (*mns*123) mutant plant that contained by necessity the MM^2^ isomer^26^. Surprisingly, this isomer did not coelute with the strange *Chlorella* Man5 (Fig. 4).

A last point worth mentioning is that the control sample already contained a small amount of a compound with just the same mass and elution position as Man5Gn (Fig. 4).

### Influence of methylation on chromatographic behavior

On the PGC columns, methylation increased retention in accordance with the view of graphitic carbon operating – at least in part – by a reversed-phase mechanism. Isobaric methyl isomers were remarkably well separated (Fig. 4). The effect on the HILIC column was opposite and more uniform. Methylation resulted in a strong forward shift roughly equivalent to one mannose residue (Supplementary Fig. 3).

## Discussion

Oligomannosidic glycans with zero to five methyl groups on terminal mannose residue constitute the N-glycomes of *Chlorella vulgaris* type strains 211-11b, SAG 211-8 l and UTEX 235. A number of commercially available algal preparations also exhibited this pattern irrespective of the species name declared by the suppliers. It must be emphasized that other products as well as other species exhibited different N-glycan patterns (Supplementary Fig. 4). These differences may harbor a valuable means for strain or species differentiation but plumbing this option would by far exceed the scope of the present study.

On a Man9 N-glycan of SAG 211-11b, MS/MS showed the methyl groups to reside on the terminal mannose residues (Fig. 4). As methylation affected glycans of all size, we propose that it takes place after the mannosidase trimming as a finishing touch of glycan maturation. The idea of incorporation of already methylated mannose residues during precursor synthesis would require that both cytosolic GDP-mannose and ER luminal dolichol-P-mannose would exist in part in a methylated version and that the respective transferases would accept these donors. Notably, *C*. *vulgaris* features 3-*O*-methyl rather than 6-*O*-methyl mannose as found for *C*. *reinhardtii*^19^. A possible purpose of methylation might be to confer resistance to unwelcome mannosidase trimming by competing organisms. In fact, it did confer resistance to jack bean α-mannosidase (Supplementary Fig. 5).

A recent work on *Chlamydomonas reinhardtii* revealed the surprising fact that the N-glycan Man5 (Man_5_GlcNAc_2_) did not have the common and expected structure (M^6^M^3^)M (see reference^25^ for explanation) but rather the isomer MM^2-2^ with an untruncated 3-arm^23^. This isomer is formed in the absence of ALG3 = Dol-P-Man:Man_5_GlcNAc_2_-PP-Dol α1,3-mannosyltransferase. As a consequence, heterologous expression of GnTI did not result in any change of the glycosylation profile^23^. While *Chlamydomonas* and *Chlorella* belong to different classes (*Chlorophyceae* and *Trebouxiophyceae*, respectively), they both find themselves in the phylum Chlorophyta/green algae and thus may share relevant features of N-glycan processing. However, while *C*. *reinhardtii* humbles itself with Man5 as the largest high-mannose glycan^19,23^, *C*. *vulgaris* presents mainly large high-mannose glycans that - apart from methylation - have the same structures as kidney bean glycans as judged from PGC-elution profiles. Man5, however, gave two peaks, one at the elution time of the common (M^6^M^3^)M and a peak eluting significantly earlier. The “linear” Man5 isomer MM^2-2^ described for *C*. *reinhardti*^23^ was isolated from an ALG3 deficient *Arabidopsis* plant and expected to coelute with this other Man5 isomer. However, it did not do so. A possible explanation that matches the effect of different mannose residues on retention could be that this Man5 peak is M^3^M^2^ the result of only partial alg12 action. The later eluting peak was readily converted to Man5Gn by GnTI and represented the classic isomer (M^6^M^3^)M. The GreenGem control sample lacking UDP-GlcNAc nevertheless already contained a trace of a substance with exactly the same m/z, elution time and also fragment spectrum as Man5Gn. The presence of Man5Gn in *C*. *vulgaris* is not unlikely given the presence of a gene of high homology to *Arabidopsis thaliana* MAGT (Uniprot # A0A2P6TDC6 _CHLSO). Compounds downstream of Man5Gn – if present - did not occur as major products, at least not under the applied growth conditions and not in the *C*. *vulgaris* lines studied. Genes/proteins with high homology to ALG3, ALG12 and ALG9 of *Arabidopsis thaliana* can be found in *Chlorella* species (Uniprot # A0A087SGA3 _AUXPR, E1Z9Y6_CHLVA or A0A2P6U3R5_CHLSO and E1ZH92_CHLVA or A0A2P6TST7_CHLSO, respectively). These proteins are responsible for the formation of the oligomannose precursor. Together with the identical elution positions of Chlorella and bean oligomannose glycans this strongly supports the idea of conserved pathway up to Man5 and very probably even Man5Gn. Highly homologous genes have recently been identified even in a red microalga^27^ and a diatom species^15^.

The current work certainly shows that microalgae, *i*.*e*. *Chlorellas*, can harbor the complete Glc_3_Man_9_ pathway as Man5 to Man9 structures indistinguishable from plant N-glycans were found. No ALG3 bottle neck exists in *Chlorella* as is the case in *C*. *reinhardtii*^23^, but *C*. *vulgaris* would have been but a slightly better choice for heterologous expression of GnTI, as only about 1% of the total N-glycome existed as the GnTI substrate (M^6^M^3^)M. The occurrence of traces of Man5Gn, *i*.*e*. the initial product of GnTI in *C*. *vulgaris* is backed up by glycoproteomic evidence for the occurrence of terminal HexNAc in an other *Trebouxiophyceae* species^20^.

Utilization of *C*. *vulgaris* as a production host for glycoproteins would at first require to identify the *O*-methyltransferase acting on terminal mannose residues. While the immunogenicity of methylated N-glycans has not yet been demonstrated, it is arguable that only methyltransferase knock-out microalgae could be considered for the expression of therapeutic glycoproteins. Such knock-out lines could also answer the scientific question as to the biological purpose of N-glycan methylation.

## Methods

### Sources of biological samples

Culture collection strains and commercial tablets as collected in 2016 to 2017 are listed in (Table 1). Live algae were grown for 15–21 days in 50 mL Bold’s Basal Medium in sterile Erlenmeyer flasks, in the presence of Tetracyclin (final concentration 10 μg/mL). Autotrophic cultivation was carried out at 22 °C, under continuous illumination by the built-in light source (Osram T8 L 36 W 830 G13 Lumilux, Munich, Germany) and shaking on 160 rpm. Microalgae concentration was determined by optical density measurement at 682 nm. Cells were harvested at the end of the exponential growth phase by centrifugation (5000 g; 15 min) and were subjected to further analysis immediately. Leaves of an *alg*3 mutant *Arabidopsis thaliana* line (*mns*123 *alg*3 quadruple knock out) were kindly provided by Richard Strasser (see Related manuscript).

**Table 1:** Origin of algae strains and products.

Strain	Location of vendor	web site	Designated as
SAG 211-11b	Göttingen, Germany	sagdb.uni-goettingen.de/	*Chlorella vulgaris*
SAG 211-8 l	Göttingen, Germany	sagdb.uni-goettingen.de/	*Chlorella vulgaris*
UTEX395	Austin, Texas	*utex*.*org/*	*Chlorella vulgaris*
**Company/Product**			
BioPure.eu Limited	Guntramsdorf, Austria	www.biopure.eu/	*Chlorella pyrenoidosa*
Taiwan Chlorella “Green Gem”	Taipei, Taiwan	www.taiwanchlorella.com/	*Chlorella pyrenoidosa*
Pure Planet Greenfoods	Vilsheim, Germany	www.pureplanet.de	*Chlorella sorokiniana*

### Extraction and fractionation of N-glycans

N-glycans were isolated by a combination of pepsin digestion, cation exchange based capturing of peptides and glycopeptides, digestion with peptide:N-glycosidase A (Europa Bioscience Ltd, Cambridge, UK), repeated cation exchange and polishing by reversed phase solid phase extraction as described albeit on smaller scale^28^.

HILIC on a TSK Amide80 column (4 × 250 mm, 5 µm; Tosoh Bioscience GmbH, Griesheim, Germany) was performed on underivatized glycans for preparative purposes^28^. Fractions of 0.5 mL were analyzed by MALDI-TOF MS. This led *inter alia* to a fraction of Me_3_Man_9_GlcNAc_2_ that could be used for ESI-MS/MS without any danger of interference by adjacent peaks with more or less methyl groups (Supplementary Fig. 3).

### Mass spectrometric methods

MALDI-TOF MS of glycan pools was performed with dihydroxybenzoic acid as the matrix on a Bruker Autoflex MALDI-TOF instrument in the positive ion reflectron mode. Usually, unreduced samples were analyzed, but in some cases reduction with 1% sodium borohydride was done to readily discriminate glycan from non-glycan peaks.

Reduced glycans were analyzed by LC-ESI-MS with a porous graphitic carbon (PGC) column (0.32 µm x 150 mm) operated by an Ultimate RSLC (Thermo Scientific, Vienna) connected to a Maxis 4 G Q-TOF MS (Bruker, Bremen, Germany)^25^. N-glycans from white kidney beans were used as reference^25^. MS/MS was performed in positive mode.

The monosaccharide constituents were analyzed after hydrolysis of glycan pools of fractions with 4 M trifluoroacetic acid at 100° for 4 h. Sugars were reduced with NaBD_4_, peracetylated and analyzed on an Agilent J&W HP-5ms GC Column (30 m x 0.25 mm, 0.25 µm) installed in a GC-MS system (GC 7820 A & MSD 5975, Agilent, Waldbronn, Germany). Partially methylated alditol acetates were available from a previous study^29^ and their relative retention times were additionally confirmed by literature data^30^.

### GlcNAc-transferase reaction

Man5 substrates were prepared from the N-glycan pools of either Green Gem tablets or kidney beans by size separation on an amide column as described^31^. Rabbit GlcNAc-transferase I (GnTI) lacking the N-terminal 105 amino acids^32^ was expressed with an N-terminal His_6_-tag using a pVT-Bac vector and the baculovirus insect cell system^33^. The enzyme was purified by metal chelate chromatography^34^. The purified enzyme was added to 0.45 nmol of Man5 from GreenGem tablets or kidney beans in 50 mM MES buffer (pH 7.0) containing 500 nmol MnCl_2_ and 10 nmol UDP-GlcNAc (Kyowa Hakko, Tokyo) and incubated overnight at 37 °C. The glycans in the mixtures were purified using carbon solid phase cartridges (Multi-Sep Hypercarb 10 mg, Thermo Scientific, Vienna) as described^35^. The eluate was dried, taken up in pure water and analyzed by PGC-LC-ESI-MS as described above.

## Supplementary information

Supplementary Figures

## Data Availability

The datasets (MS files) generated during the current study are available from the corresponding author on request.
