# Supplementary material for: N-glycans of the microalga Chlorella vulgaris are of the oligomannosidic type but highly methylated
Source: Sci Rep. 2019 Jan 23;9:331. doi: 10.1038/s41598-018-36884-1 (PMC6344472; doi:10.1038/s41598-018-36884-1)

Supplementary figures for manuscript

N-glycans of the microalga Chlorella vulgaris are of the oligomannosidic type but highly methylated

Réka Mócsai, Rudolf Figl, Clemens Troschl, Richard Strasser, Elisabeth Svehla, Markus Windwarder, Andreas Thader, Friedrich Altmann

Correspondence and requests for materials should be addressed to F.A.
(email: friedrich.altmann@boku.ac.at)

Affiliations
1 Department of Chemistry, University of Natural Resources and Life Sciences, Vienna; Muthgasse 18, 1190 Vienna, Austria

2 Department of Agrobiotechnology Tulln, University of Natural Resources and Life Sciences, Vienna; Konrad-Lorenz-Straße 20, 3430 Tulln an der Donau, Austria

3 Department of Applied Genetics and Cell Biology, University of Natural Resources and Life Sciences, Vienna; Muthgasse 18, 1190 Vienna, Austria

4 Current affiliation: Fresenius Medical Care Adsorber Tec GmbH, Magnesitstraße 9, 3500 Krems, Austria

5 Current affiliation: Shire, Process Development & Technical Services, Benatzkygasse 2-6, Vienna, Austria

6 Current affiliation: IST Austria, Am Campus 1, 3400 Klosterneuburg, Austria

Supplementary Figure 1: Positive mode MSMS spectra of Man5Gn generated by virtue of recombinant GnTI from Man5 of either white kidney beans (**A**) or a GreenGem Chlorella sample as in Fig. 4F (**B**). The MSMS spectrum of the small peak already present in the algae preparation with mass and retention time of Man5Gn is shown in panel (**C**).

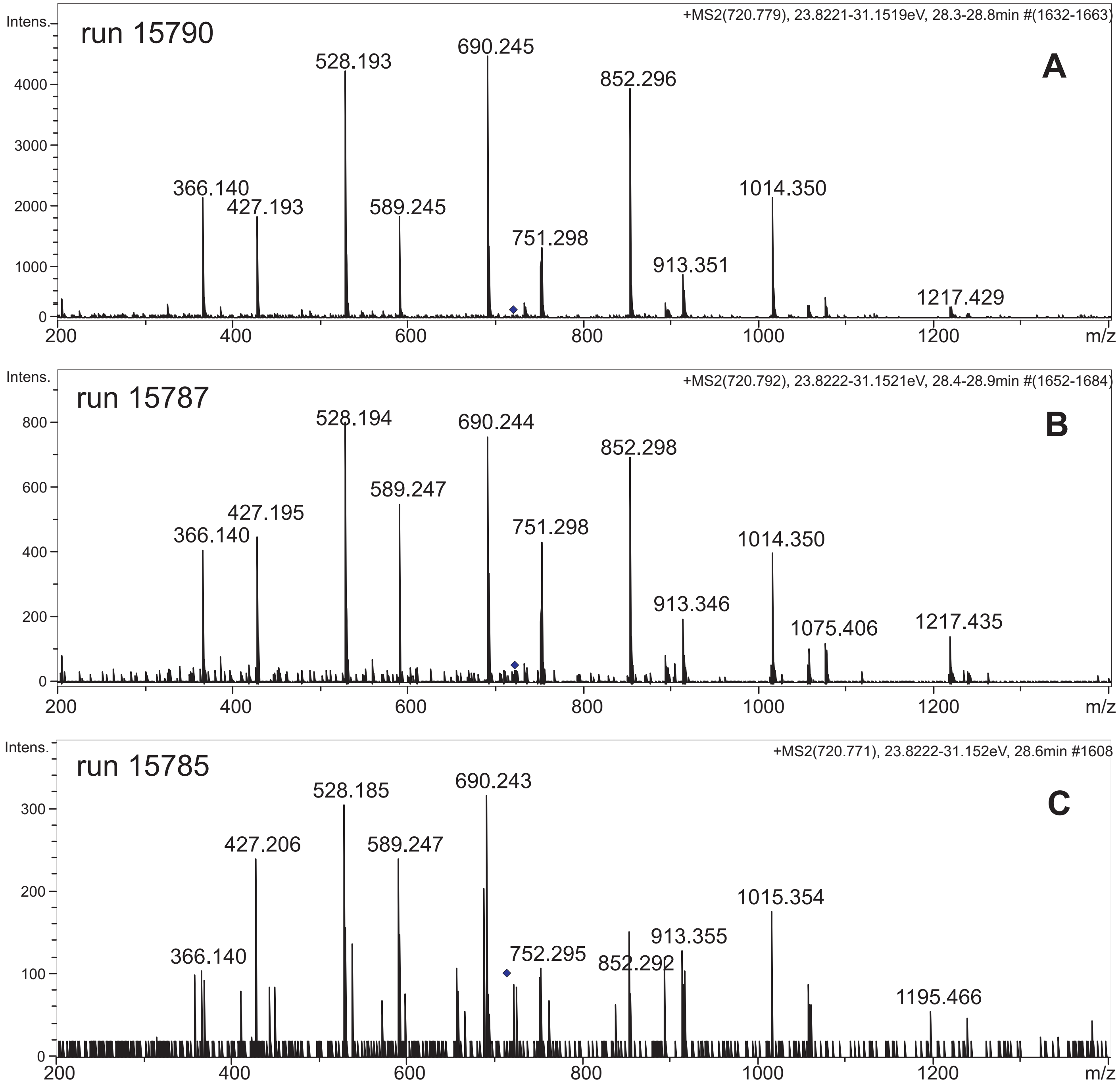

Supplementary Figure 2: Decoration of algal Man5 with GlcNAc and core fucose. A HILIC fraction enriched in Man5 (incidentially just the regular Man5 and not the earlier eluting isomer) was incubated with GnT I.

The same sample was either incubated without (trace A) and with UDP-GlcNAc (panel B).

At a later time point (hence the differing retention times), sample B was further incubated with recombinant bovine α1,6-fucosyl-transferase (expressed without the first 30 residue in Sf9 cells).

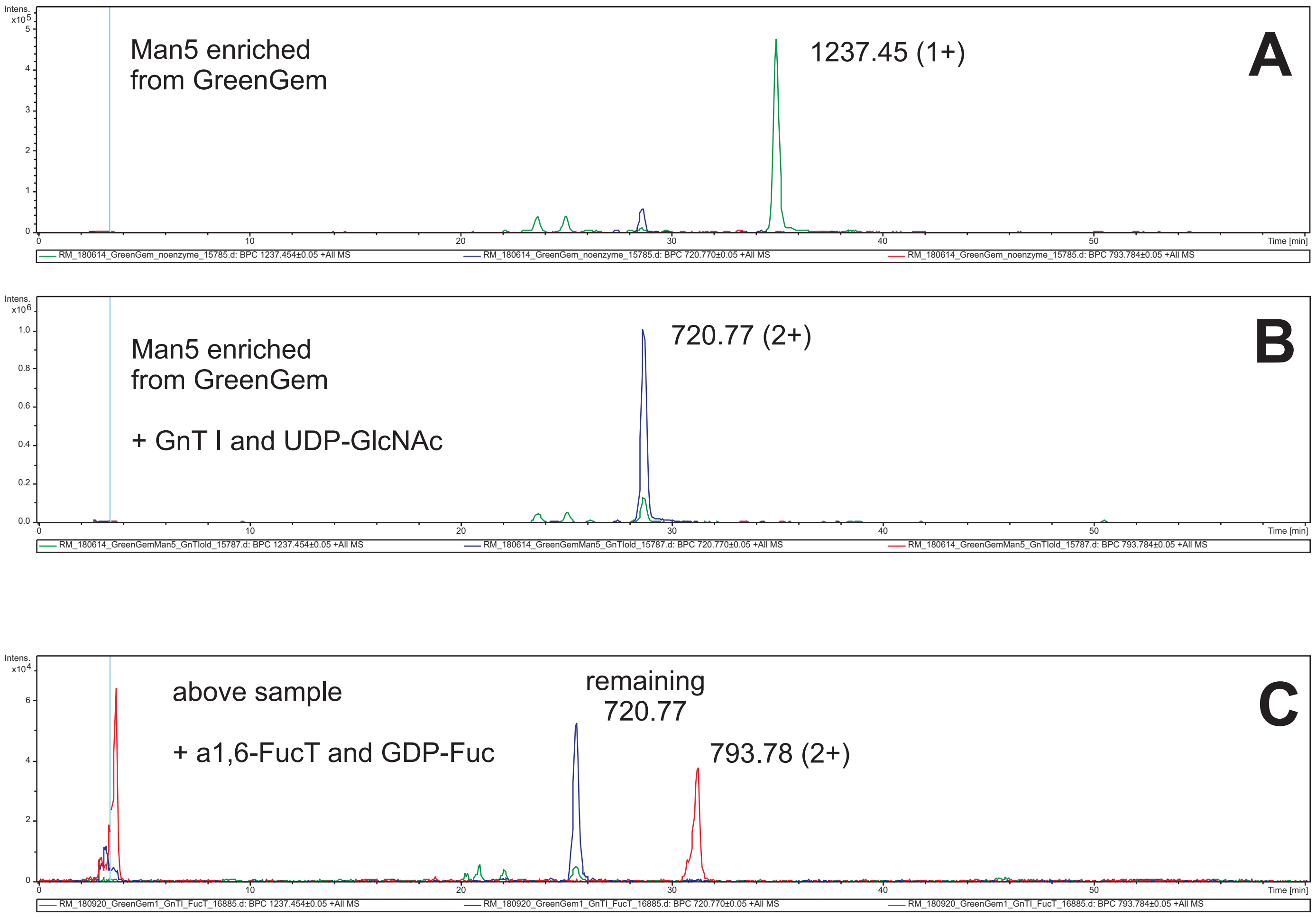

Supplementary Figure 3: Elution of glycans form GreenGem Chlorella tabs on a TSK-amide 80 HILIC column. Fractions of 0.5 mL were collected and analyzed by MALDI-TOF MS.
M9 stands for Man9 and M9-2 for a Man9 glycan with 2 methyl groups. The abbreviation is printed in bold or bold/underlined when the compound has it´s major or very major occurrence in this fraction.

45	46	47	48	49	50	51	52	53	54	55	56	57	58
		M9-5	M9-5	**M9-4**	M9-4	<u>**M9-3**</u>	M9-3	<u>**M9-2**</u>	M9-2	M9-1		M9	**M9**
		M8-4	M8-3	<u>**M8-3**</u>	M8-3	<u>**M8-2**</u>	M8-2			M8	**M8**		
		**M7-3**		M7-2				M7	<u>**M7**</u>	M7			
M6-2<br><u>**M6-3**</u>	M6-2					<u>**M6**</u>	M6						
			M5	<u>M5</u>									

Supplementary Figure 4: MALDI-TOF MS glycan profile of *Chlorella pyrenoidosa* tablets from Heidelberger Chlorella GmbH (Leiden/St. Ilgen, Germany). While oligomannosidic structures are not methylated, other species apparently containing pentose(s) and methyl groups are seen. As an example, a mass of 1329.44 Da indicates a glycan consisting of 3 hexoses, 2 HexNAcs an 3 pentoses.

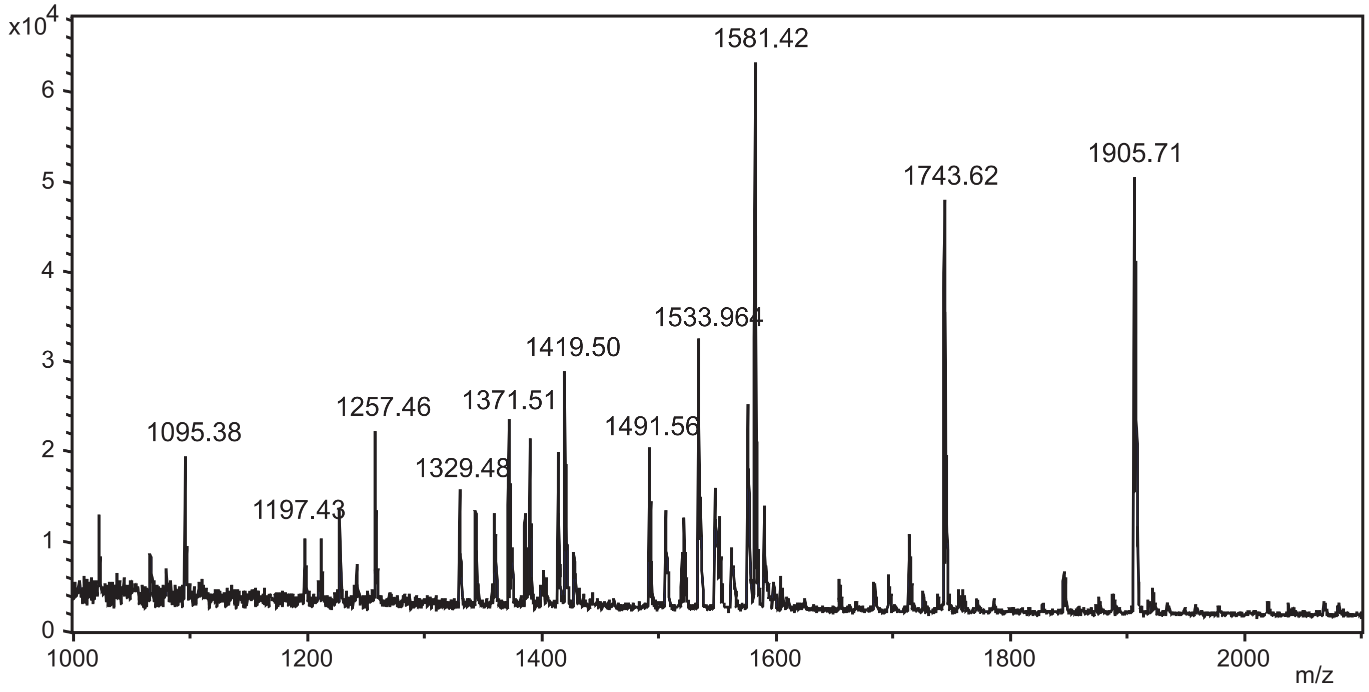

Supplementary Figure 5: Effect of jack bean α-mannosidase on methylated oligomannosidic N-glycans from C. vulgaris 211-11b. A HILIC fraction containing multiply methylated Man8 and Man9 was incubated with a dose sufficient for digestion of oligomannosidic structures from kidney bean. Apparently, the methyl groups prevent the action of the exo-glycosidase.

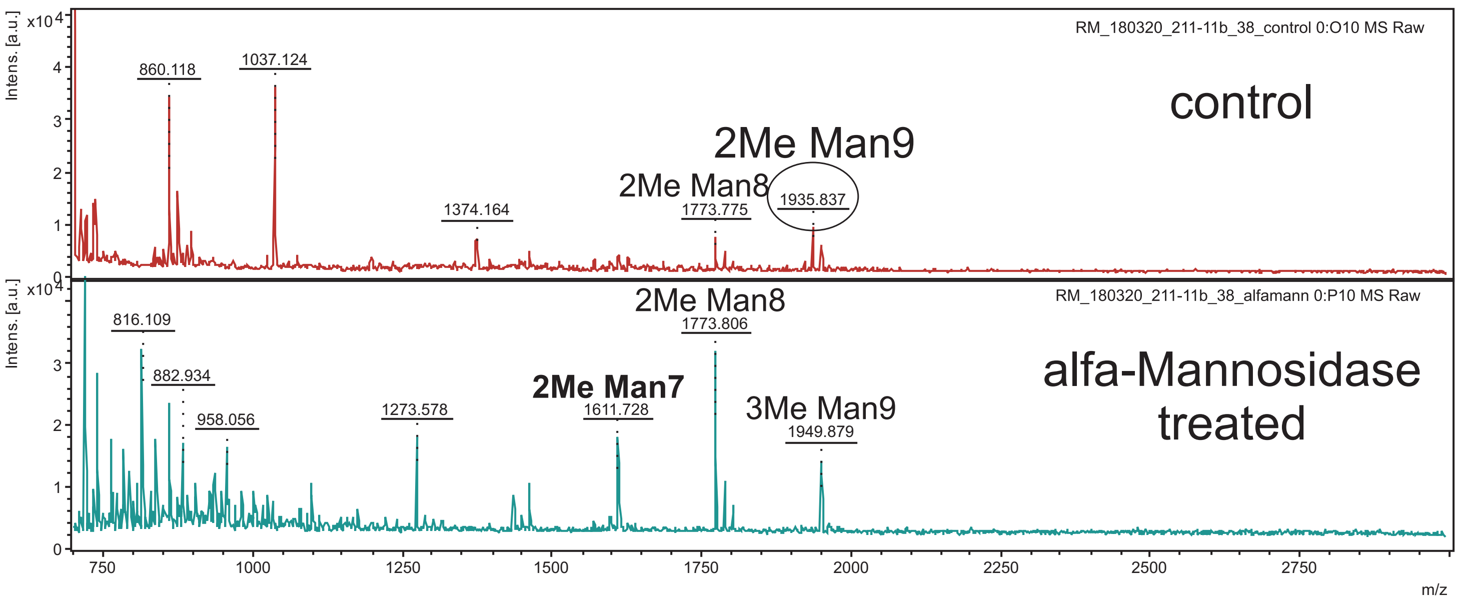

Supplement: Supplementary file 1 — Supplementary Figures [file 41598_2018_36884_MOESM1_ESM.pdf]
